# American Medical Association Notes

**Published:** 1890-07

**Authors:** 


### AMERICAN MEDICAL ASSOCIATION.

### Journalistic Echoes from the Nashville Meeting.

While the meeting may be pronounced a successful one in a general way, it was not marked by the presentation of anything new or startling in a scientific way.—*Weekly Medical Review, May 31st.*

The Nashville meeting, just closed, was a thoroughly successful one, no matter what the point from which the view may be taken. Forty States and Territories were represented — the largest number in the history of the association. It goes without saying that almost without exception the delegation from each State was composed of many of her most representative men.—*Love's Medical Mirror.*

Nashville is the title of an exquisite little brochure issued by the Lambert Pharmacal Company to the members of the American Medical Association. This souvenir contains not only much information of value, but also a number of finely executed artotypes, illustrative of points of interest in Nashville. This souvenir is so handsome that it will find a ready place in the office or parlor of every physician who is fortunate enough to obtain a copy thereof.—*St. Louis Medical and Surgical Journal, June, 1890.*

The Section work of the Association exhibited a marked improvement upon that of any previous year. There was more of scientific study and less conjecture and trash.—*American Lancet.*

The proposition to elect vice-chairmen of the sections originated with Dr. W. W. Potter, of Buffalo, chairman of the Section of Obstetrics and Diseases of Women.

Dr. W. W. Potter, of Buffalo, the president of this (Obstetrical) section, in his annual address, gave one of the best in the convention.—*Southern Medical Record.*

The meeting was a success from progressive, professional and scientific standpoints, and in its social features, perhaps, unexcelled in the history of the association. * * * Of the section work . . . it may be said that it was superior in most respects to the work of the past.—*The Medical Age.*

A NEW section was created, to be known as the Section of Materia Medica and Pharmacy. Dr. Frank Woodbury, of Pennsylvania, was named as chairman, and Dr. W. G. Ewing, of Tennessee, as secretary. Another new section, designated the Section on Dietetics and Physiology, was created.

THE PROVIDENT CHEMICAL WORKS, of St. Louis, are extensive manufacturers of phosphatic food products. They were represented by Messrs. Walter S. Burns and George P. Potee, and an attractive display of their Crystaline Phosphate. It is principally the phosphate of calcium, with the minor inorganic constituents of the tissues and phosphoric acid. It presents a pasty consistency, which is readily soluble in water, and makes an agreeable tart drink, especially when sweetened. They claim that the active properties are secured by using this crystalloid form. Samples and literature were given free to physicians.—*Dietetic Gazette.*

A NOVEL and altogether original feature of the exhibit of Messrs. Seabury and Johnson was the presentation of a fine surgical chair to the most popular physician in attendance at the convention. The contest was decided by votes of the physicians *themselves*, and the chair was awarded to Dr. Richard Douglas, an eminent physician, of Nashville.

AN ATTEMPT was again made to relegate the election of the section officers to the nominating committee. This was one of those spasmodic efforts of Keller — Keller of Hot Springs, Jim., — to bring himself forward to the notice of the house. The only wonder is that such as he are even tolerated, and how he could carry with him so large a vote is inexplicable. It is everywhere conceded that the sections were never doing as good work as now. The officers are now elected by the men who know their worth best, the test of merit being the work done in the sections and not the ability to lobby with the nominating committee. However, this effort of the lobby did not succeed, and let us hope it will not be again attempted, at least not until the present system proves a failure.

THE floor is the place from whence to carry on legislative business; so says the association by its resolution to prevent a coterie of ex-presidents from controlling affairs by swarming upon the stage and speaking therefrom to questions before the house. Verily, the day of deliverance has come.

